# Highly Efficient and Reusable Montmorillonite/Fe_3_O_4_/Humic Acid Nanocomposites for Simultaneous Removal of Cr(VI) and Aniline

**DOI:** 10.3390/nano8070537

**Published:** 2018-07-17

**Authors:** Haijiao Lu, Jingkang Wang, Fei Li, Xin Huang, Beiqian Tian, Hongxun Hao

**Affiliations:** 1 School of Chemical Engineering and Technology, Tianjin University, Tianjin 30072, China; luhaijiao@tju.edu.cn (H.L.); jkwang@tju.edu.cn (J.W.); hl509@cam.ac.uk (F.L.); x_huang@tju.edu.cn (X.H.); coolpeanut@163.com (B.T.); 2 State Key Laboratory of Chemical Engineering, Tianjin University, Tianjin 30072, China; 3 Collaborative Innovation Center of Chemical Science and Engineering, Tianjin 30072, China

**Keywords:** nanocomposites, wastewater, adsorption, kinetics, isotherms, reusability

## Abstract

Recyclable nanomaterials are in great need to develop clean technology for applications in the removal of water contaminants. In this work, easily separable montmorillonite/Fe_3_O_4_/humic acid (MFH) nanocomposites were fabricated through a facile hydrothermal route. It was found the adsorption ability and stability of MFH was significantly enhanced due to the synergistic effects between montmorillonite, Fe_3_O_4_ nanoparticles and humic acid. The MFH nanocomposites are highly efficient and recyclable as they can remove at least 82.3% of Cr(VI) and 95.1% of aniline in six consecutive runs. The adsorption mechanism was investigated by analyzing the kinetic parameters of pseudo first-order, pseudo second-order, and intraparticle diffusion models and describing the equilibrium isotherms of Langmuir and Freundlich models. Results indicated different adsorption mechanisms of Cr(VI) and aniline by MFH. The readily synthesized MFH nanocomposites can act as effective and practical materials for environmental applications.

## 1. Introduction

As one of the most toxic heavy metals and common contaminants in wastewater, Cr can be found in wastewater from painting, tanning, and metallurgical industries [1,2]. It generally exists in the form of Cr(III) or Cr(VI) with the latter hundreds of times more toxic than the former. Cr(VI) is highly hazardous to organs, such as skin, liver, and lung, and even mutagenic to organisms [3]. In addition, it causes liver damage, pulmonary congestion, and skin irritation. Converting Cr(VI) to Cr(III) is a common and efficient strategy to solve the problem of Cr(VI). Meanwhile, aniline is a group of emerging contaminants in wastewater from many industrial processes, such as painting, pharmacy, and rubber [4]. Aniline can greatly harm human and aquatic life as it is highly toxic and it can accumulate in the environment [5]. It can enter the body through inhalation, digestion and skin absorption, convert hemoglobin to methemoglobin and result in cyanosis [4]. In recent years, growing attention has been focused on aniline removal strategies. In practical situations, heavy metals like Cr(VI) and aniline can coexist in industrial wastewater [6]. Thus, it is significant to achieve simultaneous removal of Cr(VI) and aniline from industrial wastewater to prevent their damage to the ecosystem and human health.

In recent years, various techniques of removing contaminants in wastewater have been investigated, including adsorption [7,8], biodegradation [9,10], photocatalysis [11,12], and biocatalytic oxidation [13,14]. Among them, adsorption has been the most widely used and effective approach in wastewater treatment. A great variety of adsorbents (e.g., active carbon [15], metal oxides [16], polymeric resins [17], zeolites [18], clays [19] and nano zerovalent iron [20]) have been utilized to remove Cr(VI) and aniline in wastewater. The application of Fe_3_O_4_ nanoparticles (NPs) as a highly efficient adsorbent attracts great research interest due to the advantages of relatively low cost, large specific surface area and good adsorption ability.

However, the small size of Fe_3_O_4_ NPs may cause problems such as easy oxidation, aggregation and difficult separation [21]. Dispersing Fe_3_O_4_ NPs on clays like montmorillonite (Mt) is an effective and economic strategy to overcome these problems [22,23]. Like Fe_3_O_4_ NPs, Mt can also adsorb contaminants in wastewater and further enhance the adsorption ability [24,25]. What is more, the stability and dispersity of Fe_3_O_4_ NPs can be improved by coating humic acid (HA) on the surface [26,27]. HA has strong complexation ability towards heavy metals and organic pollutants, which can restrict their mobility in water [28]. Besides, both Fe_3_O_4_ and HA have been reported to effectively reduce Cr(VI) to less toxic Cr(III) in many studies, and HA coated magnetite has also been utilized for Cr(VI) adsorption and reduction (See References [29,30,31] for detailed mechanisms). Studies demonstrated that substituted phenols, α-hydroxyl carboxylic acids, oxalic acid, and α-carbonyl carboxylic acids in HA participated in the reduction reaction of Cr(VI) and lower pH can make Cr(VI) more easily reduced by improving the redox potential of Cr(VI)/Cr(III) [30,31]. However, to get better adsorption capacity, stability, dispersity, and reducibility, Fe_3_O_4_, HA, and their composite need further modification.

In this study, montmorillonite/Fe_3_O_4_/humic acid (MFH) nanocomposites were readily prepared through a simple hydrothermal route. They were obtained by coating HA on Fe_3_O_4_ NPs surface and then dispersing the particles onto Mt. Apart from the advantage of easy synthesis, MFH also possessed strong adsorption ability, good stability and high recyclability due to the synergetic effects between the components. MFH showed great promise for applications in practical contaminants removal in wastewater.

## 2. Chemicals and Methods

### 2.1. Chemicals

FeCl_3_·6H_2_O (ACS), FeSO_4_·7H_2_O (≥99.0%), ammonia solution (25%) and humic acid sodium salt (AR) were obtained from Aladdin Biochemical Technology Co., Ltd., Shanghai, China. Na-montmorillonite (cation exchange capacity of 0.9 meq∙g^−1^) was produced in Beishan, Gansu, China. K_2_Cr_2_O_7_ (≥99.95%) and hydrochloric acid (36.5–38.0 wt%) were provided by Tianjin Guangfu Fine Chemical Research Institute, Tianjin, China. 1,5-diphenylcarbazide (AR) was provided by Tianjin Chemart Chemical Technology Co., Ltd., Tianjin, China. Aniline (≥99.5%) was purchased from Shanghai Macklin Biochemical Co., Ltd., Shanghai, China. Ethanol absolute (≥99.8%) and NaOH (≥98.0%) were provided by Tianjin Real&Lead Chemical Technology Co., Ltd., Tianjin, China. Deionized water was utilized throughout the study.

### 2.2. Fabrication of MFH

**Scheme 1:**
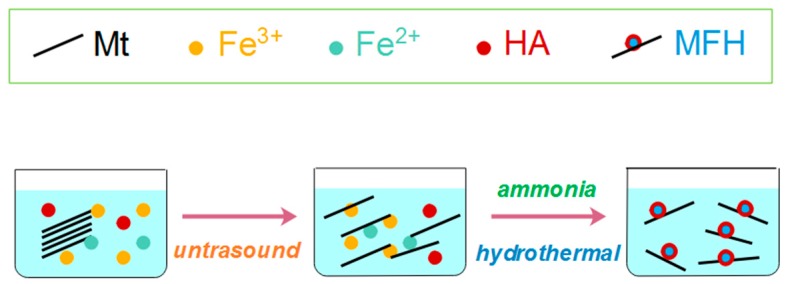
Fabrication of MFH nanocomposites.

As shown in Scheme 1, MFH nanocomposites of different Fe_3_O_4_/Mt mass ratios were obtained using a facile hydrothermal method as follows: 2.7962 g FeCl_3_·6H_2_O, 1.9580 g FeSO_4_·7H_2_O, 0.2280 g humic acid sodium salt and 1.5 g Mt (Fe_3_O_4_:Mt = 1:20)/0.75 g Mt (Fe_3_O_4_:Mt = 1:10)/0.3 g Mt (Fe_3_O_4_:Mt = 1:4)/0.075 g Mt (Fe_3_O_4_:Mt = 1:1) was dissolved in 60 mL 50% ethanol in water (*v*/*v*) and sonicated for 3 h. 4.6 mL of ammonia solution was added into the solution kept at 90 °C. Then, the suspension was transferred into a Teflon-lined autoclave (100 mL) and kept at 120 °C for 2 h. After the autoclave was cooled down naturally, the product was separated by centrifugation, washed by water and ethanol (three times each), and dried in a vacuum oven (60 °C, 6 h). For comparison, 2.7962 g FeCl_3_·6H_2_O and 1.9580 g FeSO_4_·7H_2_O was dissolved in 50% ethanol in water (*v*/*v*, 60 mL). Following the same procedure as described above, bare Fe_3_O_4_ NPs were obtained.

### 2.3. Characterization

Transmission electron microscopy (TEM) was conducted using a Tecnai G2 F20 (FEI, Eindhoven, The Netherlands) at 200 kV. The Brunauer–Emmett–Teller (BET) method was utilized to calculate the specific surface area (*S_BET_*). Nitrogen adsorption isotherms (*T* = 77 K) were obtained using an ASAP 2020 adsorption analyzer (Micromeritics, Norcross, GA, USA). Total pore volume (*V_tp_*) and average pore diameter (*D_p_*) were obtained using Barrett–Joyner–Halenda (BJH) method. A D/max-2500 X-ray diffraction analyzer (Cu Kα λ = 0.154 nm, 100 mA, 40 kV, 6° min^−1^, Rigaku, Tokyo, Japan) was used to collect X-ray diffraction (XRD) patterns. Fourier transform infrared spectroscopy (FTIR) was recorded by Tensor 27 (Bruker Optics, Ettlingen, Germany). X-ray photoelectron spectroscopy (XPS) was performed on an ESCALAB 250XI (Thermo Fisher, Waltham, MA, USA) for surface analysis. C element content was measured by TOC-VCPH (Shimadzu, Kyoto, Japan) and those of Si, Al and Fe elements were detected using inductively coupled plasma optical emission spectrometry (ICP-OES) iCAP 7400 (Thermo Fisher, Waltham, MA, USA). The content of Cr(VI) was determined using 1,5-diphenylcarbazide by a U-3010 UV-vis spectrophotometer (Hitachi, Tokyo, Japan) (λ = 540 nm) [32]. The concentration of aniline in samples was measured using a U-3010 UV-vis spectrophotometer (Hitachi, Tokyo, Japan) (λ = 230 nm) [33].

### 2.4. Batch Adsorption Experiments

One-hundred mL water with Cr(VI) (0–40.0 mg∙L^−1^) and aniline (0–100.0 mg∙L^−1^) at pH = 3.0–11.0 was added into in 250 mL flasks. The value of pH was controlled by adding 0.05 M HCl or NaOH. The solution was agitated at 400 rpm and kept at 25 °C. 0.05–0.40 g∙L^−1^ adsorbents were added in the flasks. Every 5 min during the experiment process (100 min), samples (1 mL) were taken out for UV analysis. After the nanocomposites were collected using a magnet, they were washed with 50% ethanol in water (*v*/*v*) several times and then dried. To evaluate the reusability, they were reused in the next five runs. Removal efficiency and equilibrium adsorption capacity *q_e_* (mg∙g^−1^) were obtained using Equations (1) and (2). All experiments were performed in triplicate.

(1) $$\mathrm{Removal}\;\mathrm{efficiency}=\frac{c_{0}-c_{t}}{c_{0}}\times 100\%$$

(2) $$q_{e}=\frac{(c_{0}-c_{e})V}{m}$$

*c*_0_, *c_t_* and *c_e_* (mg∙L^−1^): initial, final and equilibrium concentrations, respectively; *m* (g): the amount of adsorbent added; *V* (L): the volume of solution.

## 3. Results and Discussion

### 3.1. Characterization

**Figure 1:**
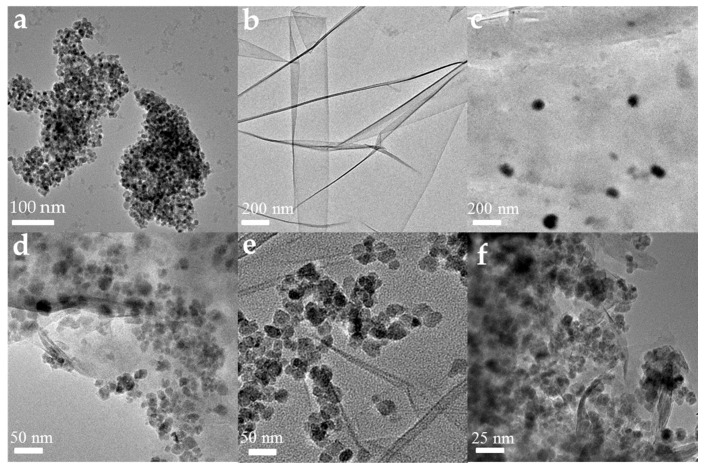
TEM of (**a**) bare Fe_3_O_4_ NPs; (**b**) Mt single layer obtained by sonication, different mass ratios of MFH nanocomposites: (**c**) Fe_3_O_4_:Mt = 1:20; (**d**) Fe_3_O_4_:Mt = 1:10; (**e**) Fe_3_O_4_:Mt = 1:4; (**f**) Fe_3_O_4_:Mt = 1:1.

As shown in Figure 1a, bare Fe_3_O_4_ NPs tended to aggregate, which would reduce specific surface area, dispersity, mobility, and adsorption ability. Following the synthesis procedure in Section 2.2, HA-coated Fe_3_O_4_ NPs were dispersed onto Mt layers to overcome this problem. After sonication, Mt of fewer layers (See Figure 1b) can be obtained, which possessed higher specific surface area than normal Mt powder. As both Mt and HA were negatively charged in the form of Na-Mt and humic acid sodium salt respectively, HA was less likely to compete with Fe_3_O_4_ NPs to bind with Mt. The electrostatic attraction between Fe_3_O_4_ NPs and Mt, and between Fe_3_O_4_ NPs and HA, along with the electrostatic repulsion between Mt and HA, functioned as the main interaction forces to disperse HA-coated Fe_3_O_4_ NPs onto Mt and maintain the structure stability. The ratio of HA-coated Fe_3_O_4_ NPs loaded on Mt was controlled by varying the ratio between Mt and Fe sources. In Figure 1c–f, different mass ratios of HA-coated Fe_3_O_4_ NPs were loaded onto Mt. According to Table 1, MFH (Fe_3_O_4_:Mt = 1:4) had the highest specific surface area (*S_BET_*) among MFH of different mass ratios. With the increase of Fe_3_O_4_:Mt ratio in MFH, the total pore volume (*V_tp_*) of MFH increased, and the average pore diameter (*D_p_*) narrowed (Fe_3_O_4_:Mt < 1:4) and then widened (Fe_3_O_4_:Mt > 1:4). Compared with MFH in Figure 1c,d, the larger *S_BET_* and *V_tp_* of MFH in Figure 1e was due to more Fe_3_O_4_ NPs loaded onto Mt. However, when Mt was overloaded with Fe_3_O_4_ NPs, the aggregation of Fe_3_O_4_ NPs would result in loss of *S_BET_* and increase of *D_p_* compared with MFH in Figure 1e. Therefore, the MFH nanocomposites investigated in this paper were prepared according to the optimum mass ratio of 1:4 (Fe_3_O_4_:Mt). The *S_BET_* and *V_tp_* of as-prepared MFH were increased significantly compared with those of Mt and bare Fe_3_O_4_ NPs. MFH enriched the pollutants by adsorption and significantly improved the removal efficiency [34,35].

**Figure 2:**
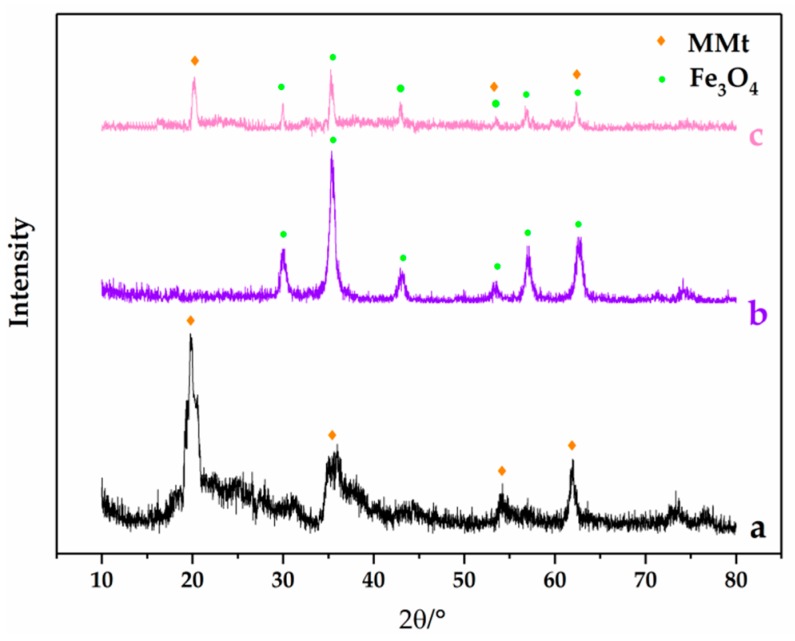
XRD patterns of (**a**) Mt; (**b**) Fe_3_O_4_ NPs and (**c**) MFH nanocomposites.

The XRD patterns of Mt, Fe_3_O_4_ NPs and MFH are shown in Figure 2. For the XRD pattern of Mt, there were four diffraction peaks at 2θ = 20.4°, 35.9°, 54.1° and 62.2°. As for bare Fe_3_O_4_ NPs, the characteristic peaks at 2θ = 30.2°, 35.8°, 43.3°, 52.6°, 57.2°, and 62.6° can be found. In the XRD pattern of MFH, the typical diffraction peaks of both Mt and Fe_3_O_4_ NPs can be seen, demonstrating their high crystallinity and the successful dispersion of Fe_3_O_4_ NPs onto Mt. Particularly, the peak of Mt at 2θ = 20.4° showed a slight shift to 2θ = 19.6° in MFH. It could be explained by that as the insertion of larger hydrolyzed iron species replaced Na^+^ in initial Mt, the planar stress increased and thus resulted in the shift to lower angle in XRD pattern [36,37]. Note that as an organic component HA does not diffract and thus cannot be distinguished by XRD.

**Figure 3:**
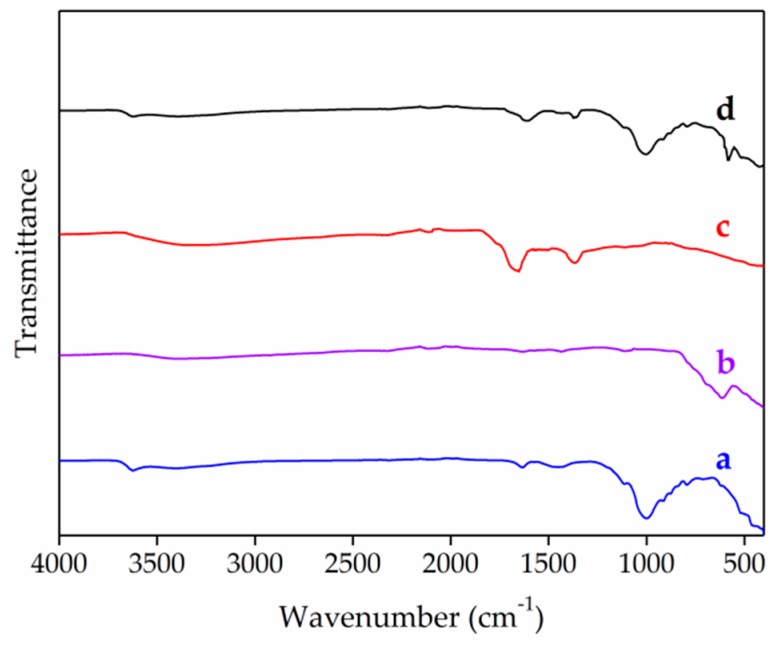
FTIR of (**a**) Mt; (**b**) Fe_3_O_4_ NPs; (**c**) HA; and (**d**) MFH nanocomposites.

To clarify the coating of HA on Fe_3_O_4_ NPs, FTIR was carried out. In Figure 3a, the band at ~1030 cm^−1^ was attributed to the stretching vibration absorption of Si-O of Mt [38]. In the Fe_3_O_4_ curve (Figure 3b), the main absorption was observed at around 582 cm^−1^, corresponding to Fe–O bending vibration [39]. In Figure 3c, the typical C=O stretching of carboxylate in HA was located at ~1680 cm^−1^, and the band (~1350 cm^−1^) can be attributed to –CH_2_– scissoring [40]. In Figure 3d, all the characteristic bands of Mt, Fe_3_O_4_ NPs and HA mentioned above can be seen, verifying the formation of MFH. The C=O stretching in MFH (featured at ~1550 cm^−1^) showed blue shift compared with that of free HA (~1680 cm^−1^). This change can also provide evidence that the carboxylate anions of HA interacted with the surface of Fe_3_O_4_ NPs.

**Figure 4:**
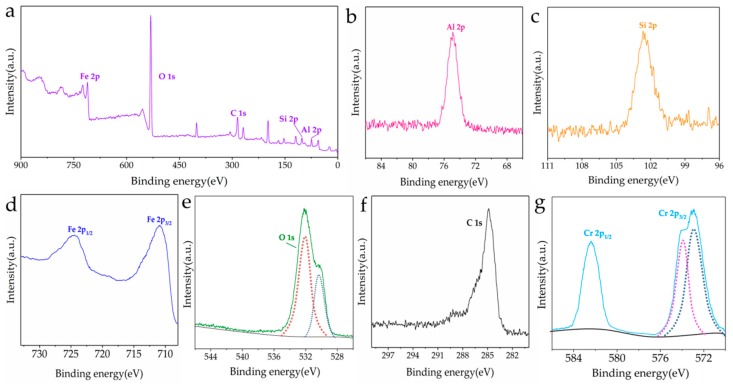
XPS of the MFH nanocomposites before (**a**–**f**) and after (**g**) removal experiments.

Besides, XPS was performed for surface composition analysis. The existence of Mt in MFH can be verified by the characteristic peaks of Al 2p and Si 2p (Figure 4b,c). Fe_3_O_4_ NPs can be confirmed by the peaks at 724.8 eV (Fe 2p_1/2_), 710.7 eV (Fe 2p_3/2_) and 530.2 eV (O 1s) (Figure 4d,e). The two split peaks of O 1s at 530.0 eV and 532.0 eV were assigned to Fe–O and hydroxyls, respectively. Besides, since spectrum of C 1s in MFH (Figure 4f) was distinguished, the existence of organic HA can also be verified. In summary, XPS indicated that the oxidation of Fe_3_O_4_ NPs was prevented by coating HA on Fe_3_O_4_ NPs surface and dispersing HA-coated Fe_3_O_4_ NPs onto Mt layers. As shown in Figure 4g, the Cr 2p_3/2_ peak of MFH after removal experiments can be split into two peaks at 574.3 and 573.2 eV (the characteristic peaks of Cr(VI) and Cr(III)), respectively, suggesting that both Cr(VI) and Cr(III) existed on MFH surface [41,42]. Therefore, it can be concluded that Cr(VI) can be reduced to Cr(III) species (e.g., Cr(OH)_3_) and precipitated on MFH surface.

The total organic carbon (TOC) content of raw HA was measured to be 43.81% (mass fraction). According to the dosage of HA used in MFH synthesis, the TOC content of as-prepared MFH was calculated to be 5.68%, which was very close to the measured value (5.34%). Besides, according to ICP-OES, the contents of Si, Al and Fe in MFH were 4.24%, 1.60% and 50.13%, respectively. As the contents of Si and Al in raw Mt were 24.18% and 8.86% respectively, theoretical contents of Si, Al and Fe in MFH were calculated to be 4.13%, 1.51%, and 49.4%, respectively. Therefore, the chemical compositions of as-prepared MFH were close to the initial dosage, which suggested the firm combination between Mt, Fe_3_O_4_ NPs and HA.

**Figure 5:**
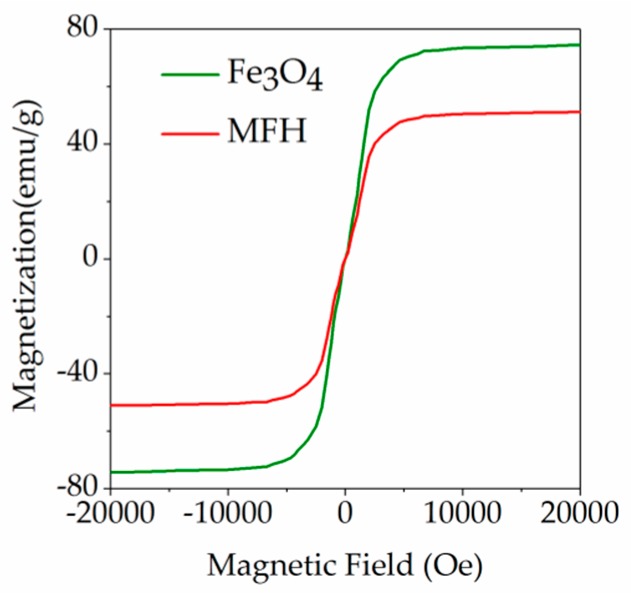
Magnetic hysteresis loops of Fe_3_O_4_ NPs and MFH nanocomposites.

To study the recyclability, magnetic hysteresis loops of MFH were plotted in Figure 5. The saturation magnetization (*Ms*) of Fe_3_O_4_ NPs and MFH were measured to be 74.3 and 51.1 emu·g^−1^, respectively. The decrease of *Ms* was resulted from the total contents of Mt and HA in MFH, which turned out to be 31.2% after calculation [28]. The composition value agreed well with that the initial dosage and calculation value of ICP-OES and TOC (30.6%). With a relatively high *Ms*, MFH can be easily separated from water by a magnet.

### 3.2. Simultaneous Removal of Cr(VI) and Aniline

**Figure 6:**
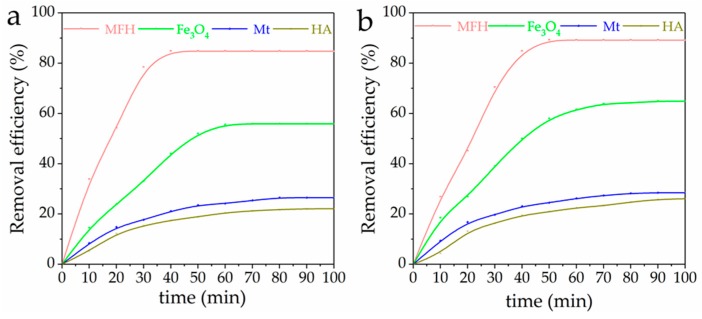
Simultaneously removing (**a**) Cr(VI) and (**b**) aniline using various adsorbents. (*c*_0_(Cr(VI)) = 40.0 mg∙L^−1^, *c*_0_(aniline) = 100.0 mg∙L^−1^, *c*(adsorbent) = 0.1 g∙L^−1^, *T* = 25 °C, pH = 3.0).

In Figure 6, the performance of MFH for removing Cr(VI) and aniline was demonstrated and compared with that of Mt, Fe_3_O_4_ NPs and HA. The removal efficiency of both Cr(VI) and aniline was MFH > Fe_3_O_4_ > Mt > HA. 84.8% of Cr(VI) and 89.2% of aniline could be removed by MFH in about 40 and 50 min, respectively. By comparing with the longer time and lower removal efficiency by Fe_3_O_4_ NPs, Mt and HA, the enhanced adsorption ability of MFH can be verified. As stated above, the specific surface area of MFH (98.86 m^2^∙g^−1^) was much larger than that of both Mt (53.72 m^2^∙g^−1^) and Fe_3_O_4_ NPs (77.24 m^2^∙g^−1^), which enriched the pollutants by adsorption and significantly improved the removal efficiency. Mt can adsorb contaminants in wastewater and further enhance the adsorption ability of Fe_3_O_4_ NPs. Coating HA onto Fe_3_O_4_ NPs greatly improved the stability and dispersity and further adsorbed more heavy metals and organic pollutants. Besides, HA also worked as reductant to reduce Cr(VI) to Cr(III) [29,30,31]. Therefore, the synergistic effects between Fe_3_O_4_ NPs, Mt and HA contributed greatly to the improvement of adsorption ability and removal efficiency in MFH.

### 3.3. Adsorption Kinetics

Adsorption kinetics helps to understand the mechanism, and the obtained data can be used to build mathematical models for interpreting interactions. To figure out the mechanisms in adsorption, pseudo first-order, pseudo second-order and intraparticle diffusion models (Equations (3)–(5)) were used for fitting the data from experiments.

(3) $$\ln(q_{e}-q_{t})=\ln q_{e}-k_{1}t$$

(4) $$\frac{t}{q_{t}}=\frac{1}{k_{2}q_{e}^{2}}+\frac{t}{q_{e}}$$

(5) $$q_{t}=k_{i}t^{1/2}+C$$

*q_e_* and *q_t_* (mg∙g^−1^): the mass of contaminants adsorbed on adsorbent at equilibrium and *t*, respectively; *k*_1_ (min^−1^), *k*_2_ (g∙mg^−1^∙min^−1^) and *k_i_* (mg∙g^−1^·min^−1/2^): the pseudo first-, second-order and the intraparticle diffusion rate constants, respectively; *C*: a constant.

These three models were tested for the adsorption of Cr(VI) and aniline on MFH in this study. The results are shown in Table 2.

Seen from Table 2, for Cr(VI), the pseudo first-order model showed the best fit and a close value of *q_e,cal_* to *q_e,exp_*. As for aniline, *R*^2^ of the pseudo second-order model (*R*^2^ = 0.982) was the highest, indicating that the adsorption of aniline by MFH followed the pseudo second-order kinetic model. It suggested that chemisorption may limit the rate of aniline adsorption. Meanwhile, the intraparticle diffusion model (*R*^2^ = 0.975) also exhibited a good fit. Therefore, the adsorption of aniline on MFH may mainly occur in two steps: first, diffusion from water to MFH surface; second, the intraparticle diffusion between the pores of MFH. Cr-bentonite [43], activated carbon [44] and multi-walled carbon nanotubes [45] with similar mechanisms to MFH can be referenced for better understanding.

### 3.4. Adsorption Isotherm

For the evaluation of adsorption capacity and the interpretation of mechanism, Langmuir and Freundlich models were utilized in this study. The former (Equation (6)) is based on the assumptions of monolayer adsorption and identical adsorption sites. The latter (Equation (7)) describes heterogeneous surface and adsorption sites [43,46].

(6) $$\frac{c_{e}}{q_{e}}=\frac{1}{bq_{m}^{}}+\frac{c_{e}}{q_{m}}$$

(7) $$\ln q_{e}=\ln K+\frac{1}{n}\ln c_{e}$$

*q_m_* (mg∙g^−1^): the maximum capacity for monolayer adsorption; *b* (L∙mg^−1^): a constant; *K* (mg^1−(1/*n*)^ L^1/*n*^∙g^−1^): a constant; n: an empirical parameter [47,48].

According to Table 3, the adsorption of Cr(VI) and aniline on MFH followed Langmuir and Freundlich models, indicating monolayer and multilayer adsorption, respectively. As displayed in Table 4, MFH possessed higher adsorption capacity towards both Cr(VI) and aniline than some reported magnetic materials [49,50,51,52].

### 3.5. Effect of pH

**Figure 7:**
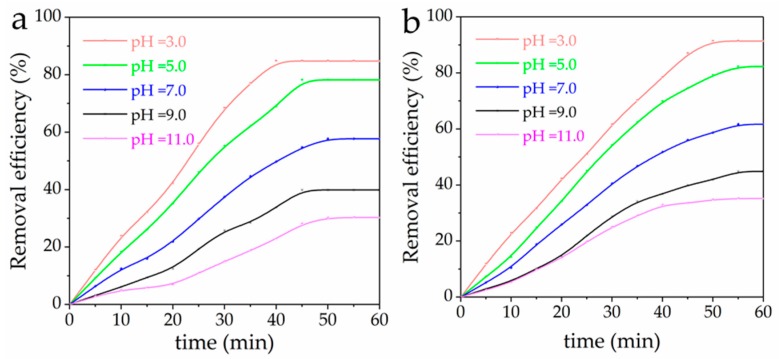
Simultaneously removing (**a**) Cr(VI) and (**b**) aniline by MFH at various pH. (*c*_0_(Cr(VI)) = 40.0 mg∙L^−1^, *c*_0_(aniline) = 100.0 mg∙L^−1^, *c*(MFH) = 0.10 g∙L^−1^, *T* = 25 °C).

Figure 7 shows that removing of both Cr(VI) and aniline was affected by pH. The adsorption rate was slowed dramatically if pH increased. Zero point charge pH (pH_ZPC_) of MFH in this study was measured to be 4.3 (±0.4) by acid–base titration method [53]. Solid surfaces of MFH had positive and negative charges when pH was higher and lower than pH_ZPC_, respectively. As the surface charge can be affected by pH, the surface of MFH will become more positively charged when pH decreases and thus will have stronger electrostatic attraction with Cr(VI) anions. Adsorption is often affected by electrostatic repulsion and attraction between adsorbent and adsorbate [30]. Stronger electrostatic repulsion due to higher pH will lead to less adsorption of Cr(VI) anions. As Equation (8) shows, studies have revealed that lower pH could enhance the redox potential of Cr(VI)/Cr(III), making Cr(VI) more easily to be reduced to Cr(III) [30]. Although unmodified Fe_3_O_4_ has reduction capacity and can reduce Cr(VI), Fe^2+^ in HA-Fe_3_O_4_ turned out not participate in Cr(VI) reduction [30,54,55]. On the contrary, higher pH leads to stronger electrostatic repulsion between them and thus the decrease of adsorption [2]. Moreover, at higher pH, the adsorption of Cr(III) species (e.g., Cr(OH)_3_) onto MFH surface reduces the adsorption ability towards contaminants [56]. On the other hand, aniline will be more protonated at lower pH (Equation (9)), which strengthens the electrostatic attraction with the negative carboxylic ions of HA in MFH. Increasing pH will significantly reduce the interaction between aniline and MFH. It can be concluded that lower pH is preferred to remove both Cr(VI) and aniline by MFH in wastewater. This tendency agrees well with previous studies on their individual removal [49,50,51,52,56].

(8) $${{\mathrm{HCrO}}_{4}}^{-}+7H^{+}+3e^{-}⇌{\mathrm{Cr}}^{3+}+4H_{2}O$$

(9) $$C_{6}H_{5}{-\mathrm{NH}}_{2}+H^{+}⇌C_{6}H_{5}{{-\mathrm{NH}}_{3}}^{+}$$

### 3.6. Effect of MFH Dosage

**Figure 8:**
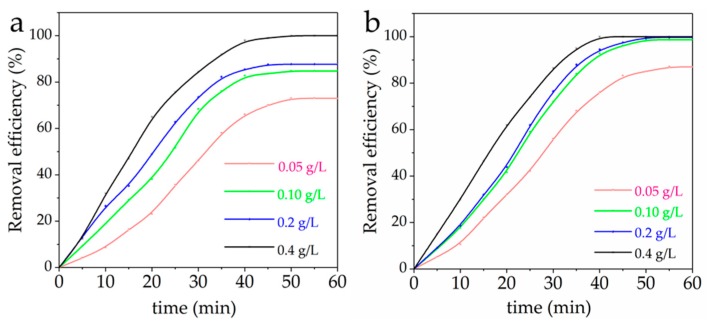
Simultaneously removing (**a**) Cr(VI) and (**b**) aniline at various MFH dosages. (*c*_0_(Cr(VI)) = 40.0 mg∙L^−1^, *c*_0_(aniline) = 100.0 mg∙L^−1^, pH = 3.0, *T* = 25 °C).

The effect of MFH dosage on the simultaneous removal of Cr(VI) and aniline was investigated, as shown in Figure 8. The results indicate that no great improvement was achieved at a higher dosage, which should provide more adsorption sites and thus contribute to contaminants removal. However, excessive MFH cannot effectively improve the adsorption efficiency due to the concentration limit of contaminants. Therefore, 0.10 g∙L^−1^ turns out to be the optimized dosage of MFH for both Cr(VI) and aniline.

### 3.7. Mutual Effect between Cr(VI) and Aniline

**Figure 9:**
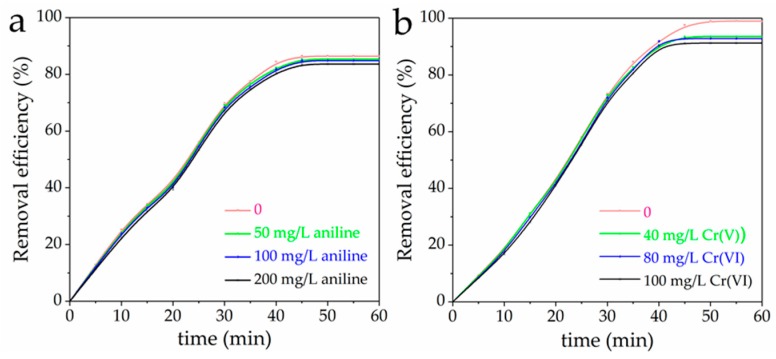
(**a**) Removing Cr(VI) with various initial concentrations of aniline by MFH. (*c*_0_(Cr(VI)) = 40.0 mg∙L^−1^, *c*(MFH) = 0.10 g∙L^−1^, pH = 3.0, *T* = 25 °C). (**b**) Removing aniline with various initial concentrations of Cr(VI) by MFH. (*c*_0_(aniline) = 100.0 mg∙L^−1^, *c*(MFH) = 0.10 g∙L^−1^, pH = 3.0, *T* = 25 °C).

Experiments were carried out to find whether Cr(VI) and aniline affected the removal of each other. As shown in Figure 9, when *c*_0_(aniline) and *c*_0_(Cr(VI)) were increased from 0 to 200 mg∙L^−1^ and 0 to 100 mg∙L^−1^ respectively, their corresponding removal efficiency barely improved. Since the upper limits of their initial concentrations were relatively high compared with actual wastewater, it can be assumed that they have no remarkable influence on the adsorption of each other by MFH. It is most likely that they are adsorbed by MFH in different mechanisms (Cr(VI), Langmuir, pseudo first-order kinetic; aniline, Freundlich, pseudo second-order kinetic), and the adsorption force is much stronger than the interaction between them.

### 3.8. Effect of NaCl Content

**Figure 10:**
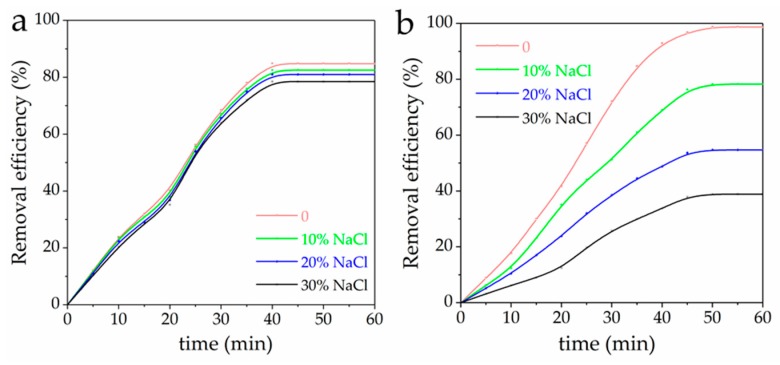
Simultaneously removing (**a**) Cr(VI) and (**b**) aniline at various NaCl contents. (*c*_0_(Cr(VI)) = 40.0 mg∙L^−1^, *c*_0_(aniline) = 100.0 mg∙L^−1^, *c*(MFH) = 0.10 g∙L^−1^, pH = 3.0, *T* = 25 °C).

Inorganic salts of high contents, especially NaCl, can also be found in actual wastewater. In general, NaCl mainly comes from the salinity in initial water or that produced by adding chemical agents in the process. Since the high salinity may have influence on the removal of contaminants in actual wastewater, the effect of NaCl was also studied. Seen from Figure 10, the efficiency of Cr(VI) removal was slightly reduced with NaCl content increasing from 0 to 30% in wastewater. Therefore, it can be concluded that NaCl in wastewater has low effect on Cr(VI) removal, which follows Langmuir monolayer adsorption. On the other hand, the removal efficiency of aniline dramatically decreased with NaCl content increasing from 0% to 30% in wastewater. Negatively charged Cl^−^ may compete with carboxylic ions of HA in MFH to bind with the protonated aniline at low pH, and positively charged Na^+^ may compete against the protonated aniline for adsorption sites. As a result, the diffusion of aniline to MFH exterior surface and even the intraparticle diffusion in pore structures is impeded. Thus, the aniline removal efficiency drops and the influence will become more significant when the content of NaCl in wastewater increases.

### 3.9. Reusability

**Figure 11:**
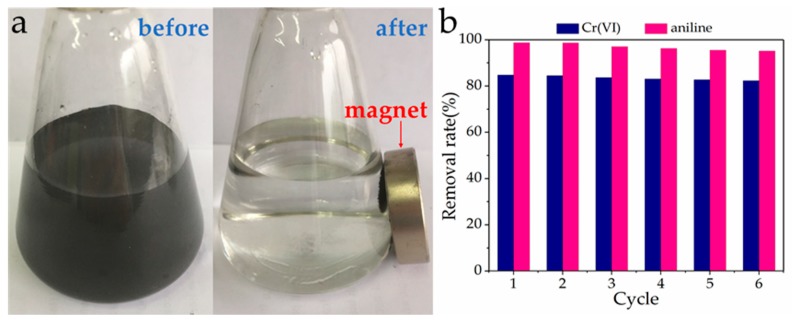
(**a**) Recycle of MFH from solution; (**b**) Reusability of MFH. (*c*_0_(Cr(VI)) = 40.0 mg∙L^−1^, *c*_0_(aniline) = 100.0 mg∙L^−1^, *c*(MFH) = 0.10 g∙L^−1^, pH = 3.0, *T* = 25 °C).

In a single run, the batch experiment was carried out as described in Section 2.4 and then the MFH were recycled from solution using a magnet, washed with 50% ethanol in water (*v*/*v*) several times and dried. The reusability was evaluated by reusing the reclaimed MFH in the next five runs. As Figure 11 shows, ≥82.3% of Cr(VI) and ≥95.1% of aniline can be removed in six consecutive cycles, demonstrating excellent reusability and structural stability.

## 4. Conclusions

In this study, montmorillonite/Fe_3_O_4_/humic acid (MFH) nanocomposites were easily fabricated through a hydrothermal route. The coating of HA onto Fe_3_O_4_ NPs and dispersion of HA-coated Fe_3_O_4_ NPs onto Mt can protect Fe_3_O_4_ NPs from oxidation and inhibit their aggregation. The synergistic effects between Mt, Fe_3_O_4_ NPs and HA turned out to effectively enhance the reactivity and efficiency of Fe_3_O_4_ NPs for the simultaneous removal of Cr(VI) and aniline from wastewater. The adsorption of Cr(VI) and aniline by MFH obey different mechanisms, Cr(VI)—Langmuir isotherm, pseudo first-order kinetic; aniline—Freundlich isotherm, pseudo second-order kinetic. Furthermore, the MFH nanocomposites possess good separation ability, stability, and reusability, making them promising materials for applications in water contaminants removal.

## Figures and Tables

**Table 1:** The specific surface area and porous features of different materials.

Material		*S_BET_* (m^2^∙g^−1^)	*V_tp_* (cm^3^∙g^−1^)	*D_p_* (nm)
Bare Fe_3_O_4_ NPs		77.24	0.24	7.5
Mt powder		53.72	0.12	8.6
MFH nanocomposites (Fe_3_O_4_:Mt, mass ratio)	1:20	62.13	0.13	8.0
	1:10	76.45	0.15	7.6
	1:4	98.86	0.19	6.5
	1:1	73.46	0.20	7.7

**Table 2:** Adsorption kinetic models using MFH at *T* = 298 K.

Model	Parameter	Cr(VI)	Aniline
	*q_e,exp_* (mg∙g^−1^)	339.25 ± 2.42	292.16 ± 2.06
Pseudo first-order	*q_e,cal_* (mg∙g^−1^)	340.31 ± 2.97	293.83 ± 2.64
	*K*_1_ (min^−1^)	0.132 ± 0.002	0.109 ± 0.001
	*R* ^2^	0.986 ± 0.001	0.911 ± 0.002
Pseudo second-order	*q_e,cal_* (mg∙g^−1^)	341.19 ± 2.80	294.22 ± 2.31
	*k*_2_ (g∙mg^−1^∙min^−1^)	0.0165 ± 0.0003	0.0274 ± 0.0005
	*R* ^2^	0.929 ± 0.001	0.982 ± 0.001
Intra particle diffusion	*k_i_* (mg∙g^−1^·min^−1/2^)	8.24 ± 0.01	11.37 ± 0.02
	*C*	0.103 ± 0.001	0.307 ± 0.002
	*R* ^2^	0.942 ± 0.001	0.975 ± 0.001

**Table 3:** Adsorption isotherm models using MFH at *T* = 298K.

Model	Parameter	Cr(VI)	Aniline
Langmuir	*q_m_* (mg∙g^−1^)	374.19 ± 3.11	393.53 ± 3.32
	*b* (L∙mg^−1^)	0.018 ± 0.001	0.096 ± 0.001
	*R* ^2^	0.995 ± 0.001	0.941 ± 0.001
Freundlich	*K* (mg^1−(1/*n*)^ L^1/*n*^ g^−1^)	0.121 ± 0.001	16.58 ± 0.09
	*n*	1.87 ± 0.01	3.74 ± 0.02
	*R* ^2^	0.907 ± 0.002	0.993 ± 0.001

**Table 4:** Adsorption capacities of various magnetic adsorbents for Cr(VI) and aniline from wastewater.

Adsorbate	Adsorbent	Adsorption Capacity (mg∙g^−1^)	Condition	Reference
Cr(VI)	Fe_3_O_4_@mTiO_2_@GO	117.94	pH = 2.0, T = 303 K	[49]
	Graphene oxide decorated with magnetic cyclodextrin	120	pH = 3.0, T = 298 K	[50]
	PPY/Fe_3_O_4_	243.9	pH = 2.0, T = 298 K	[51]
	MFH nanocomposites	374.19	pH = 3.0, T = 298 K	this study
Aniline	Fe_3_O_4_-activated carbon	90.91	pH = 6.0, T = 293 K	[52]
	Fe_3_O_4_/graphene	202.84	pH = 3.0, T = 298 K	[46]
	MFH nanocomposites	393.53	pH = 3.0, T = 298 K	this study
